# No ontogenetic shift in the realised trophic niche but in Batesian mimicry in an ant-eating spider

**DOI:** 10.1038/s41598-020-58281-3

**Published:** 2020-01-27

**Authors:** S. Pekár, L. Petráková Dušátková, C. R. Haddad

**Affiliations:** 1 Department of Botany and Zoology, Faculty of Science, Masaryk University, Kotlářská 2, 611 37 Brno, Czech Republic; 2 Department of Zoology & Entomology, University of the Free State, P.O. Box 339, Bloemfontein, 9300 South Africa

**Keywords:** Behavioural ecology, Mimicry

## Abstract

In predators an ontogenetic trophic shift includes change from small to large prey of several different taxa. In myrmecophagous predators that are also mimics of ants, the ontogenetic trophic shift should be accompanied by a parallel mimetic change. Our aim was to test whether ant-eating jumping spider, *Mexcala elegans*, is myrmecomorphic throughout their ontogenetic development, and whether there is an ontogenetic shift in realised trophic niche and their mimetic models. We performed field observations on the association of *Mexcala* with ant species and investigated the natural prey of the ontogenetic classes by means of molecular methods. Then we measured the mimetic similarity of ontogenetic morphs to putative mimetic models. We found *Mexcala* is an inaccurate mimic of ants both in the juvenile and adult stages. During ontogenesis it shifts mimetic models. The mimetic similarity was rather superficial, so an average bird predator should distinguish spiders from ants based on colouration. The realised trophic niche was narrow, composed mainly of ants of different species. There was no significant difference in the prey composition between ontogenetic stages. Females were more stenophagous than juveniles. We conclude that *Mexcala* is an ant-eating specialist that reduces its prey spectrum and shifts ant models during ontogenesis.

## Introduction

True predators capture a high number of prey items during their lifetime^1^. They typically take prey that is smaller than themselves^2^, due to a decline in capture success with relative prey/predator size^3^. As predators grow in size during ontogenesis they are able to accomplish an ontogenetic shift in prey preference by selecting prey of a larger size. This is well known for spiders, the most diversified group of terrestrial true predators, where the size of prey correlates well with the size of growing spiders^4^.

In specialised predators, trophic adaptations are often highly specialised^5^, increasing the capture efficiency of their preferred prey. In rare cases, a specialised capture strategy combined with very effective venom enables even tiny juveniles of specialised predators to catch the same prey type as adult spiders^6^. However, even specialised predators typically accomplish an ontogenetic shift in prey preference within the range of their preferred prey. Two mechanisms of prey shifting have been observed, which depend on the developmental characteristics of their prey. Specialists feeding on prey with a gradual (hemimetabolous) development (e.g., termites), switch between developmental stages of the exclusive prey, from small to large individuals^7^. Specialists preying on holometabolous insects shift between differently sized imagoes of distinct prey types^8–11^, or if imagoes of prey are polymorphic, then they shift from small to larger forms of the same prey species^12^.

A very few trophic specialists use their prey species also for a defence. This is the case in predators feeding on ants, which are also their Batesian mimics (i.e. myrmecomorphy). For example, *Aphantochilus*^13^ or *Zodarion* spiders^14^ both imitate and feed on the model ant species. If the predator is at the same time a Batesian mimic of its prey, then the ontogenetic trophic shift should be accompanied by a parallel ontogenetic Batesian mimetic shift. Myrmecomorphic species either imitate ant species only during certain ontogenetic stages^15^ or imitate different ant species during ontogenesis, i.e. transformational mimicry^16–18^. In the latter case, the mimic imitates ant models that co-occur and coexist spatially in the same microhabitat as all of the predator’s ontogenetic stages. Alternatively, the predator’s early ontogenetic stages are not or are only poorly myrmecomorphic. This is the case in juveniles of both *Aphantochilus* and *Zodarion* spiders, whose early instars captured the same ant species as adult individuals, although their mimetic similarity to their prey is rather poor^13^.

Here we focused on a spider that is specialised to catch ants presumably during its entire life cycle. *Mexcala elegans* Peckham & Peckham (shortened to *Mexcala* onwards) is a diurnally active salticid spider that appears to feed particularly on its model ant species of the genus *Camponotus*^19^. However, in the laboratory, adult individuals of this spider species subdued a range of ant species and other prey types^20^. The prey and defensive habits of juvenile individuals of *Mexcala* remain unknown. *Mexcala* shows an ontogenetic dimorphism in colouration^19^. Specifically, early instars have metallic blue-green colouration, whereas older instars have silver-grey colouration, in some cases accompanied by two pairs of yellow-orange spots on the abdomen. The colouration may have important implication on its foraging and defensive strategy.

Our aim here was to investigate whether *M. elegans* spiders are Batesian mimics of ants throughout their ontogenetic development and whether there is ontogenetic shift both in Batesian mimicry (i.e. transformational mimicry) and prey preference. For this purpose, we performed field observations on the association of *M. elegans* with putative model species, measured the mimetic similarity of major ontogenetic classes to mimetic models, and investigated the natural prey of these classes by means of molecular gut-content analysis, which is a powerful tool able to identify prey obtained via cryptic feeding^21^. We predicted that if the spider is myrmecomorphic at each ontogenetic stage then it would preferably catch its mimetic model because of similarities in body size and close spatial occurrence.

## Results

### Association with ants

Different ontogenetic stages did not show a significant association to any of six ant species (GEE-p, *χ*^2^_2_ < 5.7, P > 0.06). Overall, there were on average 1.42 (standard error (SE) = 0.19) adults, 1.33 (SE = 0.21) large juveniles and 1.30 (SE = 0.17) small juveniles of *Mexcala* per tree. The most common ants (relative frequency on 50 trees) were *C. cinctellus* (0.94, i.e. occurred on 47 trees), followed by *C*. *postoculatus* Forel (0.41), *Cataulacus* *intrudens* (Smith) ((shortened to *Cataulacus* onwards) (0.62), *Crematogaster castanea* Smith (0.29), *Atopomyrmex mocquerysi* André (0.21) and *Crematogaster* sp. (0.06).

### Phenotypic similarity

Among all the ant species co-occurring with *Mexcala* spiders, only three ant species had a similar colouration: *C. cinctellus* ants, which were black with golden or silver-shining gaster; *Cataulacus* ants, which were uniformly black; and *Polyrhachis schistacea* (Gerstäcker) ants (shortened to *Polyrhachis* onwards), which were silver-shining grey. These were selected as the putative mimetic models in further analyses.

**Figure 1:**
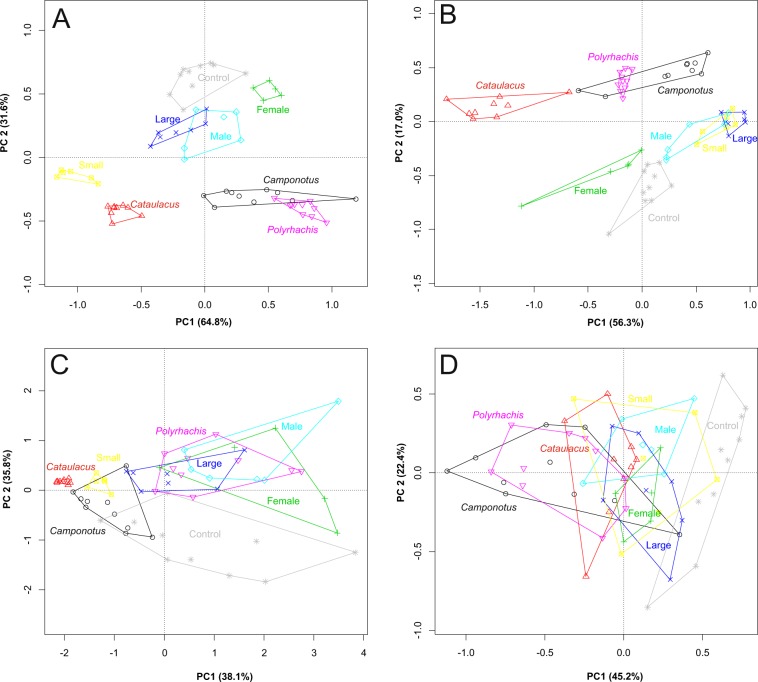
PCA ordination of body size measures (**A**), body contour (**B**), colouration (**C**), and movement (**D**). The first two axes (PC1 and 2) are shown. Control was a non-myrmecomorphic salticid spider.

The first two PCA axes of body size measures explained 96.4% of variation (Fig. 1A, Table S1). The sizes of adults and large juveniles of *Mexcala* were most similar to the control (*Stenaelurillus*), while that of small juveniles was similar to *Cataulacus* ants.

The first two PCA axes of body contour explained 73.3% of variation (Figs. 1B, S1C,D). The contours of all *Mexcala* stages were closer to the control than to the ants. All stages were similarly distant from the ant models.

The first two PCA axes of colouration explained 86.2% of variation (Figs. 1C, S1A,B). The colouration of males, females and large juveniles was similar to *Polyrhachis*, while the colouration of small juveniles was clearly different and similar to *C. cinctellus* and *Cataulacus*. Colouration of all stages was different from the control.

The first two PCA axes of movement explained 67.6% of variation (Fig. 1D, Table S2). The spiders moved in a zig-zag manner with raised forelegs. The movement of all *Mexcala* stages overlapped to a large degree, and was also similar to both all ant models and the control.

**Figure 2:**
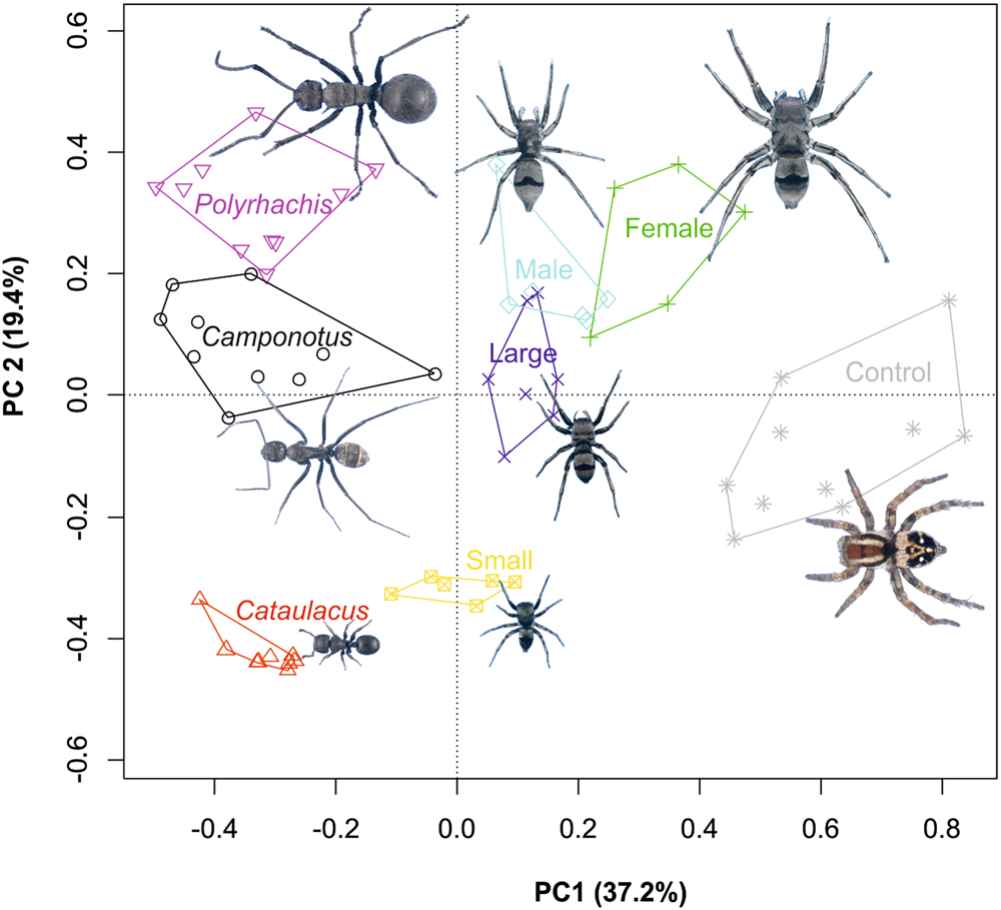
PCA ordination of original PCA scores of movement, body contour, size, and colouration. The first two axes (PC1 and 2) are shown. Control is a non-myrmecomorphic salticid spider of the genus *Stenaelurillus*. Images of spiders and ants are overlapping the particular polygons. Images of models (ants) and control are aligned horizontally; images of mimics (spiders) are aligned vertically. Photos: M. Hrušková Martišová using Stream Motion software.

The first two axes of overall PCA explained 56.6% of variation (Fig. 2). Adult males, females and large juveniles were similar to *C. cinctellus* and *Polyrhachis*, while small juveniles were similar to *Cataulacus*. There was a significant difference in the distance of mimics from their models (ANOVA, P < 0.0001): small juveniles, large juveniles and adult males were significantly closer to their models than adult females.

### Colour discrimination by predators

**Figure 3:**
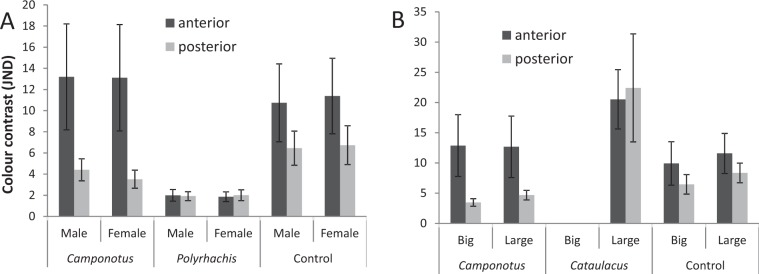
Comparison of colour contrasts of large and small juveniles (**A**) and male and female adults (**B**) of *Mexcala* with their potential ant models (*Cataulacus*, *Polyrhachis*, *Camponotus*) and a control spider (*Stenaelurillus*). Bars are means and whiskers are 95% confidence intervals.

For adult male and female *Mexcala*, the average colour contrast of the anterior and posterior body regions were far above the threshold of 3 when compared to the control and *Camponotus*, but less than 3 when compared to *Polyrhachis* (Fig. 3A). For juvenile mimics the average perceptual distances from *C. cinctellus*, *Catalulacus* and the control were above 3 (Fig. 3B). Thus, adult *Mexcala* were hardly indistinguishable from *Polyrhachis*, while juveniles were clearly distinguishable from ant models and the control.

### Realised trophic niche

We found 56 MOTUs using the ant primers (Table S3). Due to rather short length of the amplified DNA fragment, we were only able to identify prey to the genus level. We identified ten ant genera: *Camponotus* (401,460 seq.), *Atopomyrmex* (104,213 seq.), *Plagiolepis* (97,207 seq.), *Monomorium* (39,114 seq.), *Myrmicaria* (38,399 seq.), *Tetramorium* (17,919 seq.), *Pheidole* (4,498 seq.), *Cardiocondyla* (3,850 seq.), *Polyrhachis* (2,556 seq.), *Cataulacus* (149 seq.), and one MOTU that belonged to the Dipteran family Cecidomyiidae (5,450 seq.). One MOTU was similar to two different genera, *Odontomachus* and *Anochetus* (12,634 seq.). Using the general invertebrate primers, we distinguished eight MOTUs. Five of them belonged to prey, while three MOTUs belonged to the predator species. The prey was detected using those primers in one female individual and consisted of ants (*Camponotus*, Formicidae), Diptera (Cecidomyiidae) and Lepidoptera (unidentified). Overall, the breadth of the trophic niche of all stages on an order prey level was narrow, *B*_*A*_ = 0.04.

Comparison of the frequency of capture of ants with their frequency of occurrence for all spiders combined we found a significant difference (Chi-square test, ${\chi }_{3}^{2}$ = 18.2, P = 0004): *Mexcala* captured more frequently *Camponotus* and *Atopomyrmex* ants than available, but less frequently *Catalacus* and *Crematogaster* which was not found in the gut.

*Camponotus* was the most frequent prey, captured by more than 80% of ontogenetic stages. We revealed at least three different *Camponotus* species in the spiders’ guts based on a simple neighbour-joining tree reconstruction (Fig. S2). However, there might have been more *Camponotus* species, as we found no variability in the sequenced COI fragment between several *Camponotus* species that were collected as potential prey at the study site. Thus, *Camponotus* MOTU 1 was found in 92.5% of the studied spiders, in all ontogenetic stages. This species was placed close to *C. natalensis* (Smith), *C. cinctellus* and *C. rufoglaucus* (Jerdon). *Camponotus* MOTU 17 was placed close to *C. grandidieri* Forel and *C. postoculatus*, and was found in a few juveniles and subadult females. *Camponotus* MOTU 31 was most similar to *C. arminius* Forel and was only found in two spider individuals.

**Figure 4:**
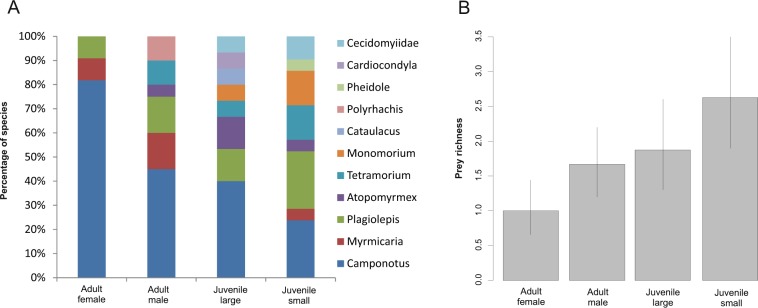
(**A**) Comparison of the proportion (percentage) of prey types for four groups identified from the gut. (**B**) Comparison of the prey richness per individual in four groups. Bars are means, whiskers are CI_95_.

While some ant genera (*Atopomyrmex*, *Plagiolepis*, *Monomorium*, *Tetramorium* and *Cataulacus*) were slightly more frequently found in the guts of juveniles than in adult and subadult individuals, comparison of the relative frequencies revealed that there was no significant difference between stages/sexes (GEE-b, *χ*^2^_30_ < 0.1, P = 1.0). Spiders fed on a variety of ant species with a similar composition. Specifically, the diet was mainly comprised of *Camponotus* (74.4%, N = 38), followed by *Plagiolepis* (28.2%), *Tetramorium* (15.4%), *Myrmicaria* (12.8%), and others (Fig. 4A). Yet, there was a significant difference between sex/stages in the proportion of individuals that tested positive for ant sequence (GEE-b, *χ*^2^_3_ = 19.4, P = 0.0002): small juveniles had the highest proportion (23.8%), followed by large juveniles (17.0%), males (15.2%), and females (9.1%). The number of prey species identified from the gut was significantly different among sex/stages (GLM-p, F_3,35_ = 5.5, P = 0.003, Fig. 4B): females had the smallest number (on average 1 prey type/specimen), while small juveniles had the largest number (≥2 prey types/specimen). The relationship between the size of the spider and the average size of its ant prey was not significant (GEE, *χ*^2^_1_ = 1.9, P = 0.17).

## Discussion

Our results show that *Mexcala* spiders are inaccurate mimics of ants. Their body contour is not antlike, but the gait is slightly similar to ants, and the body size and the colouration makes them similar to ants. Adults of *Mexcala* are not imitating one but two species of ants, *Camponotus cintellus* and *Polyrhachis schistacea*. Although we have not observed *Polyrhachis* ants to occur directly on the trunks of *Vachellia xanthophloea* trees together with *Mexcala* spiders, we found them on the foliage of neighbouring bushes and low-growing *Cissus rotundifolia* creepers, where *Mexcala* also occurs. Small juveniles of *Mexcala* were most similar to *Cataulacus intrudens*. This shows that *Mexcala* shifts from one ant model to another during ontogenetic development, but that this is not coupled with an improvement in mimetic similarity. Similar transformational mimicry has been reported for other myrmecomorphic species^15^. As all ant models co-occur, we failed to find an association of certain ontogenetic stages with only specific ant models.

In adult females, there is another colour form that is, however, rare. The colouration of this rare form is black with two pairs of orange spots on the abdomen. The most probable mimetic model of this form is nymphs of an alydid heteropteran (Pekár, unpublished), which occur directly on the trunks of *Vachellia xanthophloea* trees. These bugs were also rare, so a future study is needed to disclose the similarity and find whether the alydid bugs are noxious models.

One of the plausible hypotheses explaining the evolution and existence of inaccurate mimicry is the multimodel hypothesis^22^. In this case, inaccurate mimics imitate more than one model superficially but none accurately, and this provides the mimic with an advantage to occur on a larger spatial scale than when imitating a single model. Such mimic(s) might be engaged in a mimetic complex that includes both accurate and inaccurate mimics, possessing variety of defence levels. Recently, we discovered such a complex composed mainly of ants, but also spiders, wasps and hemipterans in Australia^23^. Indeed, it has been reported that at Ndumo there are number of ants (*Polyrhachis*, *Camponotus*), spiders (*Merenius*, *Apochinomma*, *Myrmarachne*, *Corinnomma*, *Kima*, *Castianeira*), katydid nymphs (*Eurycorypha*) and heteropterans (*Myrmoplasta*) with a similar shining-grey colouration^19,24–26^. They all may form a mimetic complex^25^. While ants and heteropterans presumably represent Mullerian mimics because they are defended by physical (*Polyrhachis*) and chemical (*Camponotus*, heteropterans) weapons, spiders and katydids are most likely Batesian mimics, as they are harmless^23^.

We have not confirmed the hypothesis of *Mexcala* being a Batesian mimic of ants experimentally using predators. The most likely predators of these spiders are wasps, mantises, birds and other spiders (Pekár, unpublished). We compared the colouration (chromatic component) of mimics and putative models using a bird visual system. This revealed that the similarity of both adult and juvenile *Mexcala* individuals to ants is on the verge of the discrimination threshold. So, an average bird should be able to distinguish them from ants. However, we have no evidence for the discrimination by other predators, such as spiders and wasps, which possess lower visual acuity than birds^27^. *Mexcala* spiders, both adult and juvenile, occurred on the tree trunks syntopically with several ant species, having same temporal activity (Pekár, pers. observation). The ants were far more abundant than the spiders, as most of them were foraging in columns. So, even though a bird predator would be able to discriminate them from ants, the spiders may be lost in the crowd of foraging ants and their chance of being captured is reduced. This is perhaps enforced by a lack of aggression on the spiders by foraging ants, enabling the spiders to move relatively freely without being detected or attacked (Haddad, pers. observation).

*Mexcala* may use aggressive mimicry, as it mainly captures ants, which is the case in a few other myrmecophagous spiders^14,28^. The superficial colouration and gait may enable spiders to approach ants without inducing an aggressive response by ants from a distance; this could also increase the chance of prey capture.

The realised trophic niche of *Mexcala* was quite narrow, supporting the former view that it is myrmecophagous. The fundamental niche was wider, as the spiders were able to catch other insect prey with a high frequency in the laboratory (namely Collembola, Isoptera, Auchenorhyncha) than those found in this study^20^. Aside from ants, Diptera and Lepidoptera sequences were only found in the guts of a few field-collected juveniles in this study, indicating that ants remain the preferred prey despite the presence of alternative food sources. As the general invertebrate primers preferentially amplify those two invertebrate orders^29,30^, we cannot exclude the possibility that *Mexcala* also fed on other prey types in the field. Although we screened the gut content of only 40 *Mexcala* individuals, obtained data clearly show that it is specialised on ants. Indeed, ants were the most common prey on the trunks (Pekár, pers. observation).

Surprisingly, the species richness of prey found in the gut of spiders was much higher in juveniles than in adults. We expected a reverse pattern, as females aim to maximise prey capture for necessary investments in production and deposition of eggs, and are therefore expected to catch a variety of ants. However, our results indicate that females instead specialise on certain prey, particularly, *C. cinctellus*. This prey type is large compared to other syntopically occurring ant species, and is probably the most profitable, though at the same time it might be more dangerous than smaller ants. Yet, once spiders possess specialised adaptations for effective capture of prey the costs related to prey capture should be negligible. Males, might kleptoparasitise on female’s and juvenile’s prey thus their prey richness was in-between those of females and juveniles^31^.

Specialised predators are characterised by a variety of prey capture adaptations^5^. Behavioural adaptations include a specific capture behaviour used against ants, which is based on a frontal approach and capture behind the head. By doing this, the spider eliminates counterattack from the prey. Metabolic adaptation, i.e. the ability to process a selected food type, has not been studied yet in this species, although venomic adaptation has. *Mexcala* has very potent venom that is more efficient on ants than on termites when compared to a closely related salticid species^32^. Its venom likely possesses specific toxins effective especially on ants^33^.

We expected to find differences in the realised trophic niche among developmental stages. Such difference has been observed in another myrmecophagous predator^12^ and is expected to eliminate intraspecific competition. In *Zodarion jozefienae* Bosmans spiders, which only feed on a highly polymorphic ant species, juveniles catch smaller morphs, adult females exploit large morphs, and adult males are kleptoparasites on their prey^31^. Such niche segregation is accomplished by the fact that males have reduced venom glands. Absence of trophic differences in *Mexcala* suggests that all developmental stages are adapted to exploit a variety of ant species.

We did not find support for a trophic shift in prey size. Small spiderlings were expected to prey on small body-sized ants, such as *Crematogaster* or *Tetramorium*, while adult specimens were expected to feed mainly on larger ants, such as *Camponotus* or *Atopomyrmex*. This was supported by the direct observations in the field^20^. However, molecular methods, which have the capacity to reveal cryptic feeding, did not support these observations. The dominant ant prey consumed by all stages was *Camponotus*. Although five genera, which were found as prey in fewer than 20% of the studied individuals, were more frequently found in juveniles than in adults, this difference was not substantial. This shows that even small juveniles are able to exploit quite large ants. Indeed, one of authors (CRH) witnessed in the field capture of large ants by juveniles. There is gathering evidence that specialised spiders in general possess the ability to catch prey larger than themselves both in juvenile and adult stage^34,35^. For example, even small instars of ant-eating *Zodarion* spiders are able to catch giant ants^6^.

In the realised trophic niche of *Mexcala* we found three prey genera of ants that could not be identified correctly – two of them do not occur in South Africa and the third one matched two genera with an identical percentage similarity. In the first two cases, we cannot exclude a possibility of contamination – the sequences have been found in almost all sequenced individuals, and the sequences formed a single MOTU. However, in each PCR set up, several negative controls were included to reveal potential sources of contamination. We never detected any positive band in the negative controls using gel electrophoresis. However, no negative control sample was sequenced together with the pooled predator samples, and thus very weak contamination could not be detected. Another possible explanation is that the sequences belonged to some inconspicuous species with a cryptic way of life that could be overlooked during sampling the potential prey.

We conclude that *Mexcala* is a specialised myrmecophagous predator that is able to exploit a range of ant species (and sizes) over the course of its ontogenetic development. We failed to find trophic shift in this species. *Mexcala* is an inaccurate myrmecomorphic species that shifts ant mimetic models during ontogenesis. Thus in myrmecophagous predators ontogenetic trophic shift might not be correlated with shift in ant-mimicry due to sympatric co-occurrence of several ant species.

## Methods

### Association with mimetic models

Preliminary observations revealed that *M. elegans* spiders forage during the morning, mainly on the trunks of trees but occasionally also on low foliage or the ground. So, in the field we selected 50 *Vachellia xanthophloea* trees at Nyamiti Pan in the Ndumo Game Reserve, South Africa, in December and March. In the morning (between 8 am and 12 am) of five sunny days we performed a visual search (in total 80 hours) and recorded the frequency of ontogenetic stages (adults, large and small juveniles) of *Mexcala* and the presence of putative ant models on the tree trunks. As putative ant models we selected such species which were (1) of similar size as *Mexcala* instars, (2) foraging upon trunk surface, and (3) more abundant than *Mexcala*. There were six such ant species: *C. cinctellus* (Gerstäcker), *C*. *postoculatus* Forel, *Cataulacus intrudens*, *Crematogaster castanea*, *Atopomyrmex mocquerysi*, and *Crematogaster* sp. Five persons were surveying each trunk for approximately 15 min. Each trunk was surveyed only once per day.

The occurrence of different stages of *Mexcala* were related to the occurrence of six most abundant ant species by means of generalised estimating equations with Poisson distribution (GEE-p) from the geepack^36^ within R^37^. GEE is an extension of GLM for correlated data^38^ and was used in order to take into account joined occurrence of different stages of *Mexcala* on the same tree (i.e. nested observations). The linear predictor included the tree as a grouping variable, presence of six ants as binary explanatory variables, and stage as a factor. The working correlation structure was exchangeable.

### Phenotypic similarity

We quantified the movement pattern, morphology (body size, body contour), and colouration of small juveniles (N = 6), large juveniles (N = 7), adult males (N = 6) and adult females (N = 5) of *Mexcala*; *C. cinctellus* (N = 10), *Polyrhachis schistacea* (N = 10) and *C. intrudens* ants (N = 9); and *Stenaelurillus guttiger* (Simon) spiders as a control (N = 10). Juvenile stages of *Mexcala* were split into two size categories, small (total body size: 2.4–4.0 mm) and large (4.6–5.8 mm), adults into males (5.3–7.1 mm) and females (6.6–7.5 mm). The small category presumably included the 2^nd^ and the 3^rd^ instars, while the large category included the 4^th^ and 5^th^ instars (Pekár, pers. observation).

We recorded the movement patterns of mimics and models using a video camera (Canon Legria HF R606). First, we placed an individual into a white plastic container (14 × 22 × 5 cm). To prevent their escape, we applied a thin layer of butter on the walls. We recorded the continuous movement of each individual for 1–2 minutes.

The video recordings of the movements were then processed using Ethovision XT software (version 11, Noldus Information Technology) to quantify the movement pattern. We estimated 11 parameters: the average and maximum velocity (cm/s); average and total meander (degree/cm) (i.e. amount of turning per unit distance); proportion of movement (%) (i.e. cumulative duration of moving) and no-movement (%); mean of relative turn angle (degree); mean of relative angular velocity (degree/s); total distance moved (cm); mean of body mobility (%); and average acceleration (cm/s^2^).

Then we killed all mimics and models by exposing them to ethyl acetate for few minutes. Freshly killed specimens were then mounted on blue sticky tape in a natural position. The blue colouration was used to allow the software (see below) to extract the image mask more easily.

We took three pictures of every individual from the dorsal view using an Olympus X12 stereomicroscope with an Olympus SC50 camera; these pictures were then combined into one image using Stream Motion software (version 1.9.4, Olympus). We illuminated specimens from the lateral sides using two fluorescent bulbs (13-W daylight ReptiGlo 2.0 UVB) with a similar light spectrum to natural light.

We then analysed the images using custom-made image analysis software^39^ to obtain data on body sizes and contours. When assessing the body contour, we straightened the binary mask of each specimen according to the body axis, measured the length of the body axis, placed 40 evenly distributed points along the body axis relative to each specimen’s body length, and measured the distance from each of these points to the body edge (excluding the legs). The software also estimated the index of circularity as another measure of body contour. Body size included the average thickness of each of three leg segments (femur, tibia, metatarsus) and the total body length. The colouration was estimated as a reflectance of the anterior and posterior body regions (see below).

First, all variables describing movement, size, contour and colouration were subjected to separate principal component analyses (PCAs). We used PCA in order to reduce the variation in multivariate space to two orthogonal dimensions. PCA was suitable as the measurements were continuous and assumed to come from the normal distribution. Within each analysis input variables were standardised. Then we extracted scores for the first two axes of the four trait groups, which explained the large majority of variation, and subjected them to the final PCA. In this analysis, all variables were standardised. From the final PCA we extracted scores along the first two axes and computed Euclidean distances between mimics and their models. These were then compared among sex/stages with a permutation ANOVA from the lmPerm package^40^ within R.

### Colour discrimination by birds

In order to find whether potential predators could discriminate mimics from models based on their colouration we measured the reflectance of spiders and ants using an Ocean Optics USB4000 spectrometer connected to an Ocean Optics PX-2 pulsed Xenon light source, which emits light in the range of 220–750 nm. The reflectance values were relative to a white standard (PTFE optical diffuser reflecting >98% along the entire wavelength range, Ocean Optics WS-1). A black standard was obtained by blocking the light resource. The optic fibre was 400 nm in diameter and was 1 cm above the subject positioned at a 60° angle. We took two measurements of each spider and ant individual, one of its anterior (head + thorax or prosoma) and one of the posterior (gaster or abdomen) (Fig. S1A,B). The measurements were then smoothed and negative values were removed using functions from the pavo package^41^ within R.

We tested whether the differences in colouration between mimics and models would be detectable by a bird predator. Since all mimetic species were collected on the bark of trees, we used the reflectance of the trunk as background. For the illumination spectrum, we used the blue sky setting because the trunks were on the forest margin exposed to direct sun light. A perception of the “average bird” predator from the vismodel function was selected for modelling because the spiders can potentially fall prey to many bird species as is the case of other salticid species^42^. For each individual spectrum, we calculated excitation values for the four photoreceptors using the vismodel function from the pavo package. Then, we estimated the perceptual distances (colour contrast) for chromatic component between mimetic spiders (small juveniles, large juveniles, adult males, adult females) and model ants using the coldist function. According to Vorobyev *et al*.^43^ birds can discriminate colour above a perceptual distance of >1 JND.

### Realised trophic niche

Besides observation of prey captured in the field, we identified the consumed prey by means of gut-content analysis. In total, 12 adult females, 12 adult males, eight large juveniles and eight small juveniles were used for gut analysis. The numbers are not high because the spiders were quite are. DNA was isolated from the legs of *Mexcala* and bodies of all potential prey collected at the study site (Table S4) using the DNeasy Blood & Tissue Kit (Qiagen), according to the manufacturer’s protocol for small tissue volumes. The cytochrome c oxidase gene was amplified using LCO1490 and HCO2198 primers^44^ and sequenced on an ABI Prism 3130 Genetic Analyzer (Applied Biosystems). Sequences are deposited in the GenBank database (accession numbers: MH673867, MK591886-MK591933). The COI sequences served as a reference for the prey assignment and for the primer design.

Prey DNA was isolated from spider opisthosomas, which were crushed and incubated overnight with Proteinase K at 56 °C, then the same protocol as mentioned above was used. Two different primer sets were used to amplify the prey DNA: the ant specific primers (ZodFormF: 5′-TTTATTAATRAWGGAGYAGGAACAGG and ZodFormR: 5′-CCTAARATTGAAGATATWCCTGCAAT^45^), and general invertebrate primers (ZBJArtF1c: 5′-AGATATTGGAACWTTATATTTTATTTTTGG-3′ and ZBJArtR2c: 5′-WACTAATCAATTWCCAAATCCTCC^46^). A specific blocking oligo with a C3 spacer modification at the 3′end, which helped to reduce the predator’s DNA amplification, was used together with the general invertebrate primers. The PCR reaction mixture consisted of 10.6 μL of Multiplex PCR Master Mix, 1.8 μL of Q-Solution, 2.8 μL of RNase-free water, 0.5 μL of 10 μM forward and 0.5 μL of reverse primers, 1 μL of 100 μM blocking oligo, and 7 μL of DNA. In each PCR performance, a negative control was included. The PCR conditions were as follows: initial denaturation at 95 °C for 15 min; 42 cycles of 94 °C for 30 s, annealing temperature (48 °C when using universal primers, 50 °C when using ant primers) for 90 s, 72 °C for 90 s, and a final extension at 72 °C for 10 minutes. The PCR primers were tagged with MID identifiers (10 bases long) and each sample was PCR amplified using primers with a unique combination of MIDs on the forward and on the reverse primer. This allowed us to assign all DNA reads to each individual predator. PCR products were detected by electrophoresis in 2% GoodView-stained agarose gels and purified using QIAquick PCR Purification Kit (Qiagen). Enrichment (emPCR) and sequencing on the Ion Torrent platform with an Ion 318 chip and 400-base read length chemistry was provided by the Centre de Recerca en Agrigenòmica (Bellaterra, Spain)^34^.

The sequencing output was processed using the Galaxy platform (https://usegalaxy.org/), BioEdit 7.2.5^47^, fastx-toolkit and the EMBOSS packages^48^. The data were filtered for quality with a Phred Q score value 20. The reads were split according to their MID combinations, resulting in files corresponding to predator individuals. MIDs were removed and too short and too long reads were excluded. The reads were collapsed and rare haplotypes (containing <2 identical reads) were removed. Sequences were translated to amino acids according to the invertebrate mitochondrial genetic code, and those containing stop codons were excluded, as they represented nonsensical sequences. Sequences with insertions and deletions causing reading frameshifts were also removed. The remaining sequences were clustered into MOTUs (= molecular operational taxonomic units) using swarm^49^ with a 4-bp cut-off (corresponding to 3% of the sequence divergence). The MOTUs that contained fewer than 10 sequences (corresponding to 0.001% of the total valid reads) were removed as potentially erroneous reads.

Each MOTU was compared to the GenBank database (https://blast.ncbi.nlm.nih.gov/Blast.cgi) using megablast, to the BOLD database (https://www.boldsystems.org/), and to the sequences obtained from the potential prey. As we were not able to reliably identify all MOTUs, we constructed simple neighbour-joining phylogenetic trees using MEGA6^50^ with all MOTUs found and the potential prey and predator sequences. The distances between the MOTUs and reference sequences allowed us to better assign ambiguous MOTUs to a taxonomic level.

We transformed the number of reads for each individual spider to qualitative data (using presence/absence coding). These data were then subjected to GEE with binomial errors (GEE-b) to compare the frequency of capture of different prey species among the ontogenetic stages. GEE was used due to nested design of several prey types within a single individual. Exchangeable correlation structure was used. To compare the species richness in the gut we used GLM with Poisson errors (GLM-p)^51^. Overdispersion was resolved by using quasipoisson setting. To test whether the body size of spiders is related to the size of captured ants we used average total body size values for each ant species, as the species show a low level of size polymorphism (Pekár, pers. observation). We used GEE with normal errors to test this hypothesis in order to account for the nested observations: several prey types within each individual. The working correlation structure was exchangeable. The standardized Levin’s index (*B*_*A*_) of niche breadth^52^ was used to calculate the fundamental trophic niche breadth.

## Data repository

Sequences are deposited in GenBank. Other data can be found at https://www.sci.muni.cz/zoolecol/inverteb/?page_id=18.

## Supplementary information

Supplementary Information.

## References

[1] Begon, M., Mortimer, M. & Thompson, D. J. *Population ecology: a unified study of animals and plants* (John Wiley & Sons, 2009).

[2] Griffiths D (1980). Foraging costs and relative prey size. Am. Nat..

[3] Nakazawa T, Ohba S, Ushio M (2013). Predator-prey body size relationships when predators can consume prey larger than themselves. Biol. Lett..

[4] Turner M (1979). Diet and feeding phenology of the green lynx spider, *Peucetia viridans* (Araneae: Oxyopidae). J. Arachnol..

[5] Pekár S, Toft S (2015). Trophic specialisation in a predatory group: the case of prey-specialised spiders (Araneae). Biol. Rev..

[6] Pekár S, Šedo O, Líznarová E, Korenko S, Zdráhal Z (2014). David and the Goliath: potent venom of an ant-eating spider (Araneae) enables capture of a giant prey. Naturwissenschaften.

[7] Eberhard WG (1991). *Chrosiothes tonala* (Araneae, Theridiidae): a web-building spider specializing on termites. Psyche.

[8] Heller, G. Zur Biologie der ameisenfressenden Spinne *Callilepis nocturna* Linnaeus 1758 (Aranea, Drassodidae). PhD thesis (Johannes Gutenberg-Universitat, Mainz, 1974).

[9] Eberhard WG (1980). The natural history and behavior of the bolas spider *Mastophora dizzydeani* sp. n. (Araneidae). Psyche.

[10] Cooper D, Williamson A, Williamson C (1990). Deadly deception. GEO.

[11] Yeargan KV, Quate LW (1997). Adult male bolas spiders retain juvenile hunting tactics. Oecologia.

[12] Pekár S, Bilde T, Martišová M (2011). Intersexual trophic niche partitioning in an ant-eating spider (Araneae: Zodariidae). PLoS One.

[13] Castanho LM, Oliveira PS (1997). Biology and behaviour of the neotropical ant-mimicking spider *Aphantochilus rogersi* (Araneae: Aphantochilidae): nesting, maternal care and ontogeny of ant-hunting techniques. J. Zool..

[14] Pekár S, Král J (2002). Mimicry complex in two central European zodariid spiders (Araneae: Zodariidae): how *Zodarion* deceives ants. Biol. J. Linn. Soc..

[15] McIver JD, Stonedahl G (1993). Myrmecomorphy: Morphological and behavioral mimicry of ants. Annu. Rev. Entomol..

[16] Mathew AP (1934). The life-history of the spider (*Myrmarachne plataleoides*) (Cambr.) a mimic of the Indian red ant. J. Bombay Nat. Hist. Soc..

[17] Edmunds M (1978). On the association between *Myrmarachne* spp. (Salticidae) and ants. Bull. Br. Arachnol. Soc..

[18] Ceccarelli FS, Crozier RH (2007). Dynamics of the evolution of Batesian mimicry: molecular phylogenetic analysis of ant-mimicking *Myrmarachne* (Araneae: Salticidae) species and their ant models. J. Evol. Biol..

[19] Wesołowska W, Haddad CR (2009). Jumping spiders (Araneae: Salticidae) of the Ndumo Game Reserve, Maputaland, South Africa. Afr. Invertebr..

[20] Pekár S, Haddad CR (2011). Trophic strategy of ant-eating *Mexcala elegans* (Araneae: Salticidae): Looking for evidence of evolution of prey-specialization. J. Arachnol..

[21] Symondson WOC (2002). Molecular identification of prey in predator diets. Mol. Ecol..

[22] Edmunds M (2000). Why are there good and poor mimics?. Biol. J. Linn. Soc..

[23] Pekár S, Petráková L, Bulbert MW, Whiting MJ, Herberstein ME (2017). The golden mimicry complex uses a wide spectrum of defence to deter a community of predators. eLife.

[24] Haddad CR, Louw SvdM (2012). A redescription of Merenius alberti Lessert, 1923 (Araneae: Corinnidae), with remarks on colour polymorphism and its relationship to ant models. Afr. Invertebr..

[25] Haddad, C. R. *Advances in the systematics and ecology of African Corinnidae spiders (Arachnida: Araneae), with emphasis on the Castianeirinae*. PhD thesis (University of the Free State, Bloemfontein, 2012).

[26] Haddad CR (2013). A revision of the ant-like sac spider genus *Apochinomma* Pavesi, 1881 (Araneae: Corinnidae) in the Afrotropical Region. J. Nat. Hist..

[27] Land, M. F. & Nilsson, D. E. *Animal Eyes* (Oxford University Press, Oxford, 2012).

[28] Oliveira PS, Sazima I (1985). Ant-hunting behaviour in spiders with emphasis on *Strophius nigricans* (Thomisidae). Bull. Br. Arachnol. Soc..

[29] Brandon-Mong GJ (2015). DNA metabarcoding of insects and allies: an evaluation of primers and pipelines. Bull. Entomol. Res..

[30] Piñol J, Mir G, Gomez-Polo G, Agustí N (2015). Universal and blocking primer mismatches limit the use of high-throughput DNA sequencing for the quantitative metabarcoding of arthropods. Mol. Ecol. Resour..

[31] Martišová M, Bilde T, Pekár S (2009). Sex-specific kleptoparasitic foraging in ant-eating spiders. Anim. Behav..

[32] Pekár S, Líznarová E, Bočánek O, Zdráhal Z (2018). Venom of prey-specialised spiders is more toxic to their preferred prey: A result of prey-specific toxins. J. Anim. Ecol..

[33] Pekár S (2018). Z. Venom gland size and venom complexity – essential trophic adaptations of venomous predators: A case study using spiders. Mol. Ecol..

[34] Michálek O, Petráková L, Pekár S (2017). Capture efficiency and trophic adaptations of a specialist and generalist predator: a comparison. Ecol. Evol..

[35] García LF, Viera C, Pekár S (2018). Comparison of the capture efficiency, prey processing, and nutrient extraction in a generalist and a specialist spider predator. Sci. Nat..

[36] Yan J, Fine JP (2004). Estimating equations for association structures. Statistics Med..

[37] R Core Team. R: A language and environment for statistical computing. R Foundation for Statistical Computing, Vienna, Austria, https://www.R-project.org/ (2017).

[38] Pekár S, Brabec M (2018). Generalized estimating equations: A pragmatic and flexible approach to the marginal GLM modelling of correlated data in the behavioural sciences. Ethology.

[39] Ježek, J. *Similarity measurement of spiders and ants*. MSc Thesis (Masaryk University, Brno, 2015).

[40] Wheeler, B. & Torchiano, B. lmPerm: Permutation tests for linear models. R package version 2.1.0. https://CRAN.R-project.org/package=lmPerm (2016).

[41] Maia R, Eliason CM, Bitton P-P, Doucet SM, Shawkey MD (2013). pavo: an R Package for the analysis, visualization and organization of spectral data. Methods Ecol. Evol..

[42] Gajdoš, P. & Krištín, A. Spiders (Araneae) as bird food. In *Proceedings of the 16th European Colloquium of Arachnology* (ed. Zabka, M.) 91–105 (Siedlce, 1997).

[43] Vorobyev M, Osorio D, Bennett A, Marshall N, Cuthill I (1998). Tetrachromacy, oil droplets and bird plumage colours. J. Comp. Physiol. A.

[44] Folmer O, Black M, Hoeh W, Lutz R, Vrijenhoek R (1994). DNA primers for amplification of mitochondrial cytochrome c oxidase subunit I from diverse metazoan invertebrates. Mol. Mar. Biol. Biotechnol..

[45] Pekár S, Petráková L, Šedo O, Korenko S, Zdráhal Z (2018). Trophic niche, capture efficiency, and venom profiles of six sympatric ant-eating spider species (Araneae: Zodariidae). Mol. Ecol..

[46] Zeale MRK (2011). for DNA barcoding arthropod prey in bat faeces. Mol. Ecol. Res..

[47] Hall TA (1999). BioEdit: a user-friendly biological sequence alignment editor and analysis program for Windows 95/98/NT. Nucleic Acids Symp. Ser..

[48] Rice P, Longden I, Bleasby A (2000). EMBOSS: The European molecular biology open software suite. Trends Genet..

[49] Mahé F, Rognes T, Quince C, de Vargas C, Dunthorn M (2014). Swarm: robust and fast clustering method for amplicon-based studies. PeerJ.

[50] Tamura K, Stecher G, Peterson D, Filipski A, Kumar S (2013). MEGA6: Molecular evolutionary genetics analysis version 6. Mol. Biol. Evol..

[51] Pekár, S. & Brabec, M. *Modern Analysis of Biological Data. Generalised Linear Models in R* (Masaryk University Press, Brno, 2016).

[52] Hurlbert SH (1978). The measurement of niche overlap and some relatives. Ecology.

