# Supplementary material for: No ontogenetic shift in the realised trophic niche but in Batesian mimicry in an ant-eating spider
Source: Sci Rep. 2020 Jan 27;10:1250. doi: 10.1038/s41598-020-58281-3 (PMC6985134; doi:10.1038/s41598-020-58281-3)
Supplement: Supplementary file 1 — Supplementary Information. [file 41598_2020_58281_MOESM1_ESM.docx]

**Table S1.** Total body length and the width of proximal parts of appendages (antennae and legs) [mm]. Means and SEs are shown.

|  | | **Total** | | | **Appendage** | | | | |
| --- | --- | --- | --- | --- | --- | --- | --- | --- | --- |
| **Species** | | **body** | | | **1** | | **2** | **3** | **4** |
| *Camponotus* | | 8.22 (0.400) | | | 0.17 (0.012) | | 0.44  (0.022) | 0.33  (0.019) | 0.35 (0.020) |
| *Cataulacus* | | 4.67 (0.106) | | | 0.11 (0.004) | | 0.14  (0.009) | 0.15 (0.003) | 0.18 (0.006) |
| *Polyrhachis* | | 9.86 (0.166) | | | 0.20 (0.003) | | 0.33  (0.005) | 0.31 (0.007) | 0.33 (0.006) |
| Small juvenile | | 2.85 (0.229) | | | 0.17 (0.005) | | 0.16  (0.006) | 0.15 (0.007) | 0.18 (0.009) |
| Large juvenile | | 5.12 (0.192) | | | 0.35 (0.016) | | 0.33  (0.017) | 0.33 (0.018) | 0.36 (0.014) |
| Adult male | | 6.08 (0.267) | | | 0.40 (0.025) | | 0.37  (0.027) | 0.37 (0.024) | 0.41 (0.021) |
| Adult female | | 7.07 (0.148) | | | 0.53 (0.014) | | 0.50  (0.013) | 0.49 (0.004) | 0.55 (0.009) |
| *Stenaelurillus* | | 4.82 (0.181) | | | 0.42 (0.012) | | 0.39  (0.008) | 0.43 (0.012) | 0.41 (0.018) |
|  |  | |  |  | |  |  |  |  |

**Table S2.** Overview of seven movement variables. Means and SEs are shown.

| **Species** | **Mean velocity**  **[cm/s]** | **Mean**  **meander [deg/cm]** | **Mobility [%]** | **Moving**  **[%]** | **Turn angle**  **[deg]** | **Angular velocity**  **[deg/s]** | **Acceleration**  **[cm/s^2^]** |
| --- | --- | --- | --- | --- | --- | --- | --- |
| *Camponotus* | 2.80 (0.37) | 195 (324) | 35.2 (3.4) | 49.5 (6.7) | 0.45 (0.35) | 11.3 (8.8) | -0.11 (0.03) |
| *Cataulacus* | 1.55 (0.11) | 554 (1066) | 21.5 (1.5) | 38.1 (3.4) | -0.46 (0.46) | -11.5 (11.5) | -0.03 (0.01) |
| *Polyrhachis* | 2.52 (0.27) | -150 (111) | 27.3 (2.1) | 52.9 (5.8) | 0.27 (0.46) | 6.9 (11.6) | -0.07 (0.01) |
| Small juvenile | 1.31 (0.41) | -266 (858) | 14.1 (2.7) | 21.2 (6.8) | -0.63 (0.89) | -15.8 (22.3) | -0.02 (0.16) |
| Large juvenile | 1.36 (0.24) | 98 (657) | 14.6 (1.3) | 21.4 (4.4) | 0.21 (0.68) | 5.43 (17.1) | 0.03 (0.05) |
| Adult male | 1.55 (0.26) | -2677(1330) | 15.9 (1.8) | 27.5 (3.5) | -0.64 (0.37) | -16.1 (9.2) | -0.01 (0.01) |
| Adult female | 1.35 (0.13) | 239 (934) | 12.9 (1.0) | 26.1 (4.3) | 0.67 (0.65) | 16.9 (16.2) | -0.04 (0.03) |
| *Stenaelurillus* | 0.42 (0.07) | -2633 (2134) | 3.9 (0.7) | 3.5 (0.9) | 0.14 (0.56) | 3.6 (14.2) | -0.39 (0.17) |

|  |  |  |  |
| --- | --- | --- | --- |
|  |  |  |  |
|  |  |  |  |
|  |  |  |  |

**Table S3.** List of DNA sequences of prey and the predator detected from the gut of *Mexcala elegans*. M = male (adult or subadult), F = female (adult or subadult), LJ = large juvenile, SJ = small juvenile. **Total** = number of sequences without stop codons and reading frame shifts, appearing more than once; **ant primer** = ant prey genera and number of prey sequences amplified with ant specific primers; **other primer** = other prey sequences amplified with general invertebrate primers (Zeale et al. 2011); **# seq.** = number of prey sequences in each MOTU per individual; **# predator seq.** = number of predator sequences. MOTUs represented by less than 0.001% of the total number of valid sequences were excluded. Prey genera represented by less than 0.5% of the valid sequences obtained from an individual spider (for ant prey only) are in violet.

| **Sex/stage** | **Total** | **Ant primer** | **# seq.** | **Other primer** | **# seq** | **# predator seq.** |
| --- | --- | --- | --- | --- | --- | --- |
| M | 3548 | *Myrmicaria*  *Plagiolepis*  Formicidae | 703  45  710 | - | - | 2088 |
| M | 9850 | *Myrmicaria*  *Cataulacus*  *Camponotus* sp.1  *Plagiolepis*  *Polyrhachis*  Formicidae | 6314  21  12  8  2  1033 | - | - | 2460 |
| M | 67171 | *Polyrhachis*  *Camponotus* sp.1  *Cataulacus*  *Plagiolepis*  *Myrmicaria*  *Monomorium*  *Atopomyrmex*  *Pheidole*  Formicidae | 861  376  118  38  41  34  14  2  65674 | - | - | - |
| M | 10073 | *Camponotus* sp.1 | 3937 | - | - | 6136 |
| M | 32971 | *Camponotus* sp.1  Cecidomyiidae  Formicidae | 2881  4  17281 | - | - | 12803 |
| M | 26319 | *Camponotus* sp.1  *Tetramorium*  Cecidomyiidae  Formicidae | 2250  288  5  10438 | - | - | 13338 |
| M | 82668 | *Camponotus* sp.1  *Plagiolepis*  *Tetramorium*  Formicidae | 56635  85  2  2281 | - | - | 23664 |
| M | 4974 | *Camponotus* sp.1  *Plagiolepis*  Formicidae | 4856  72  46 | - | - | - |
| M | 43461 | *Atopomyrmex*  *Myrmicaria*  *Plagiolepis*  *Atopomyrmex*  *Camponotus* sp.1 Formicidae | 14317  11023  1908  572  56  1252 | - | - | 14319 |
| M | 9829 | *Camponotus* sp.1  *Atopomyrmex*  Formicidae | 9398  2  429 | - | - | - |
| M | 10208 | *Tetramorium*  *Camponotus* sp. 1  *Atopomyrmex*  *Camponotus sp. 3*  *Polyrhachis*  Formicidae | 1456  377  19  2  2  8340 | - | - | - |
| M | 12559 | *Polyrhachis*  *Camponotus* sp.1  *Monomorium*  Formicidae | 1679  1433  2  4230 | - | - | 5215 |
| F | 57875 | *Myrmicaria*  *Camponotus* sp.1  *Atopomyrmex*  *Plagiolepis*  *Monomorium*  *Pheidole*  *Tetramorium*  *Cardiocondyla*  *Polyrhachis*  *Tetramorium*  Formicidae | 19994  268  89  55  41  23  14  3  2  2  37377 | - | - | - |
| F | 19157 | *Camponotus* sp.1  *Atopomyrmex*  *Pheidole*  Formicidae | 19008  3  2  144 | - | - | - |
| F | 116173 | *Camponotus* sp. 1  *Camponotus* sp. 2  *Atopomyrmex*  Formicidae | 115749  34  6  382 | - | - | - |
| F | 3554 | *Camponotus* sp.1  Formicidae | 984  575 | - | - | 1987 |
| F | 5308 | *Camponotus* sp.1  *Plagiolepis*  Formicidae | 2407  17  2 | - | - | 2882 |
| F | 7608 | *Camponotus* sp.1  *Polyrhachis*  Formicidae | 4276  2  6 | - | - | 3324 |
| F | 9493 | *Camponotus* sp.1 | 3964 | - | - | 5529 |
| F | 25790 | *Monomorium*  *Cardiocondyla*  *Camponotus* sp.1  *Myrmicaria*  Formicidae | 17296  3772  4  2  121 | - | - | 4593 |
| F | 8775 | *-* | - | - | - | 8775 |
| F | 36732 | *Camponotus* sp.1  *Pheidole*  *Atopomyrmex*  Formicidae | 30737  4  2  240 | *Camponotus*  Formicidae  Lepidoptera  Cecidomyiidae  Insecta unident. | 26  22  7  5  2 | 5687 |
| F | 4877 | *Camponotus* sp. 1  *Camponotus* sp. 3 Formicidae | 3302  47  1528 | - | - | - |
| F | 7019 | *Camponotus* sp. 2  *Camponotus* sp. 1  *Plagiolepis*  *Monomorium*  Formicidae | 5253  126  4  2  1634 | - | - | - |
| LJ | 20695 | *Plagiolepis*  *Camponotus* sp.1  Formicidae | 20641  31  23 | - | - | - |
| LJ | 32941 | *Camponotus* sp.1  *Plagiolepis*  *Tetramorium*  *Atopomyrmex*  Formicidae | 32714  92  31  2  102 | - | - | - |
| LJ | 73297 | *Atopomyrmex*  *Camponotus* sp.1 Formicidae | 63577  1759  7961 | - | - | - |
| LJ | 67136 | *Camponotus* sp.1  *Plagiolepis*  *Atopomyrmex*  *Cardiocondyla*  Formicidae | 67101  12  3  2  15 | - | - | - |
| LJ | 27305 | *Camponotus* sp.1  *Plagiolepis*  Formicidae | 6666  4  9284 | - | - | 11351 |
| LJ | 3711 | *Camponotus* sp.1  *Plagiolepis*  Formicidae | 3682  7  16 | - | - | - |
| LJ | 48596 | *Monomorium*  Cecidomyiidae  *Camponotus* sp. 1  *Tetramorium*  *Camponotus* sp. 2  *Catalaucus*  Formicidae | 341  340  186  131  20  2  47576 | - | - | - |
| LJ | 15245 | *Camponotus* sp. 1  *Tetramorium*  *Catalaucus*  *Monomorium*  *Camponotus* sp. 2  *Pheidole*  Formicidae | 9696  3033  318  49  5  2  2142 | - | - | - |
| SJ | 64914 | *Plagiolepis*  *Camponotus* sp.1  *Atopomyrmex*  Formicidae | 61963  246  14  2691 | - | - | - |
| SJ | 30515 | *-* | - | - | - | 30515 |
| SJ | 10034 | *Pheidole*  *Monomorium*  *Plagiolepis*  *Pheidole*  *Camponotus* sp.1  *Polyrhachis*  *Myrmicaria*  *Cataulacus*  Formicidae | 4469  460  446  179  81  4  2  2  4391 | - | - | - |
| SJ | 9411 | *Atopomyrmex*  *Plagiolepis*  *Tetramorium*  *Myrmicaria*  *Camponotus* sp.1 Formicidae | 2550  1547  1316  77  24  1295 | - | - | 2600 |
| SJ | 21524 | Cecidomyiidae  *Camponotus* sp.1  *Atopomyrmex*  *Tetramorium*  Formicidae | 4217  163  5  2  5164 | - | - | 11973 |
| SJ | 33066 | *Monomorium*  *Tetramorium*  *Camponotus* sp.1  *Atopomyrmex*  *Camponotus* sp. 2  *Plagiolepis*  *Catalaucus*  Cecidomyiidae  Formicidae | 26370  378  239  18  14  4  2  2  6037 | - | - | - |
| SJ | 31546 | *Monomorium*  *Plagiolepis*  *Camponotus* sp.1  *Tetramorium*  *Polyrhachis*  Formicidae | 11826  9983  183  36  8  194 | - | - | 9319 |
| SJ | 22734 | *Tetramorium*  *Camponotus* sp.1  Cecidomyiidae  *Plagiolepis*  *Atopomyrmex*  *Monomorium*  *Polyrhachis*  Formicidae | 11230  10269  882  224  8  2  2  117 | - | - | - |
| Total | 1128662 |  | 949969 | 62 |  | 178558 |

**Table S4**. List of potential prey taxa collected at the study site (arranged by order and family) in which COI gene was sequenced and used as a reference to assign the prey sequences detected in the predator guts to a taxonomic level. Access numbers are provided.

| **Order** | **(Sub)Family** | **Species** | **Access number** |
| --- | --- | --- | --- |
| \| Collembola \|  \| \| --- \| --- \| | Entomobryidae | Entomobryidae sp. | MK591924 |
| \| Archaeognatha \|  \| \| --- \| --- \| | Meinertellidae | Meinertellidae sp. | MK591925 |
| \| Thysanura \|  \| \| --- \| --- \| | Lepismatidae | Lepismatidae sp. | MK591926 |
| Orthoptera | Mogoplistidae | *Cycloptiloides* sp. 1 | MK949520 |
| Blattodea | Ectobiidae | Ectobiidae sp. | MK591927 |
| Mantodea | Mantidae | Mantinae sp. | MK591928 |
|  | Hymenopodidae | *Otomantis* sp. | MK591929 |
|  |  | *Oxypilus* sp. | MK591930 |
| Isoptera | Rhinotermitidae | *Odontotermes badius* (Haviland) | MK591921 |
|  | Termitidae | *Macrotermes natalensis* (Haviland) | MK591923 |
|  |  | *Schedorhinotermes lamanianus* (Sjöstedt) | MK591922 |
| Hemiptera | Rhyparochromidae | *Poeantius nigropictu*s (Stal) | MK591920 |
|  | Reduviidae | Harpactorinae sp. | MK591931 |
| Thysanoptera | Phlaeothripidae | *Ophthalmothrips* sp. | MK591932 |
| Coleoptera | Staphylinidae | Aleocharinae sp. | MK591933 |
|  | Cicindelidae | *Dromica tenella* Peringuey | MK591919 |
| Hymenoptera | Formicidae |  |  |
|  | Dorylinae | *Dorylus helvolus* (Linneaus) | MK591908 |
|  | Ponerinae | *Anochetus natalensis* Arnold | MK591891 |
|  |  | *Bothroponera kruegeri* (Forel) | MK591915 |
|  |  | *Euponera aenigmatica* (Arnold) | MK591898 |
|  |  | *Odontomachus troglodytes* Santschi | MK591903 |
|  |  | *Paltothyreus tarsatus* (Fabricius) | MK591899 |
|  | Pseudomyrmecinae | *Tetraponera* *natalensis* (F. Smith) | MK591910 |
|  | Myrmicinae | *Atopomyrmex mocquerysi* André | MK591897 |
|  |  | *Cataulacus intrudens* (Smith) | MK591890 |
|  |  | *Crematogaster castanea* Smith | MK591886 |
|  |  | *Crematogaster* sp. | MK591889 |
|  |  | *Monomorium* sp. | MK591896 |
|  |  | *Myrmicaria natalensis* (F. Smith) | MK591893 |
|  |  | *Nesomyrmex* sp. | MK591902 |
|  |  | *Ocymyrmex fortior* Santschi | MK591904 |
|  |  | *Tetramorium* sp. 2 | MK591888 |
|  |  | *Tetramorium* sp. 3 | MK591894 |
|  |  | *Tetramorium weitzeckeri* Emery | MK591895 |
|  | Formicinae | *Camponotus arminius* Forel | MK591918 |
|  |  | *Camponotus braunsi* Mayr | MK591887 |
|  |  | *Camponotus cinctellus* (Gerstäcker) | MK591892 |
|  |  | *Camponotus grandidieri* Forel | MK591917 |
|  |  | *Camponotus natalensis* (F. Smith) | MK591909 |
|  |  | *Camponotus petersii* Emery | MK591914 |
|  |  | *Camponotus postoculatus* Forel | MK591912 |
|  |  | *Camponotus rufoglaucus* (Jerdon) | MK591913 |
|  |  | *Camponotus* sp. 2 | MK591907 |
|  |  | *Camponotus* sp. 3 | MK591905 |
|  |  | *Lepisiota* sp. | MK591906 |
|  |  | *Polyrhachis arnoldi* Forel | MK591916 |
|  |  | *Polyrhachis gagates* (F. Smith) | MK591901 |
|  |  | *Polyrhachis schistacea* (Gerstäcker) | MK591900 |

**Fig. S1**. Summary of traits used for the similarity analysis. Smoothed curves of reflectance (A, B). Contour curves of body profile halves (C, D).

**Fig. S2.** Neighbour-joining tree of COI gene fragments of ten *Camponotus* species compared with three MOTUs assigned to *Camponotus*.
